# Investigating the efficacy of melatonin, topical sodium citrate, and multivitamin with zinc as a potential treatment for postinfectious loss of smell^☆^

**DOI:** 10.1016/j.bjorl.2024.101496

**Published:** 2024-08-31

**Authors:** Ellen Cristine Duarte Garcia, Letícia Ribeiro Rosa, Ana Caroline Rodrigues dos Santos, Gabrieli Kaori Alves Ishimatsu, Natália Medeiros Dias Lopes, Marco Aurélio Fornazieri

**Affiliations:** a Universidade Estadual de Londrina (UEL), Departamento de Clínica Cirúrgica, Londrina, PR, Brasil; b Pontifícia Universidade Católica do Paraná, Departamento de Medicina, Curitiba, PR, Brazil; c Universidade de São Paulo, Departamento de Otorrinolaringologia, São Paulo, SP, Brazil; d University of Pennsylvania, Perelman School of Medicine, Smell and Taste Center, Department of Otorhinolaryngology, Head and Neck Surgery, Philadelphia, PA, United States

**Keywords:** Zinc, Smell, Anosmia, Melatonin, COVID-19

## Abstract

**Objectives:**

Upper respiratory tract infections, including COVID-19, are associated with olfactory dysfunction and there is a need for novel therapeutic approaches. The aim of study was evaluating the effectiveness of adding melatonin, multivitamin and sodium citrate to olfactory training for the treatment of olfactory loss caused by COVID-19.

**Methods:**

We evaluated olfactory function using University of Pennsylvania Smell Identification Test (UPSIT ®) scores and self-reported patient outcomes in post-infectious smell loss cases. We investigated the effectiveness involved olfactory training combined with sodium citrate, melatonin, and multivitamin supplements with zinc over a three-month period compared to an olfactory training alone.

**Results:**

A total of 66 patients were included, with 33 in each group. There was no significant difference in the proportion of participants who showed improvement in UPSIT scores between the groups (OR = 1.43, 95% CI 0.43–4.8, *p* =  0.56). Both groups showed improvement in average test scores, but there were no significant differences in self-reported olfactory ability or discomfort with olfactory loss. Qualitative symptoms, such as parosmia and phantosmia, were reported by a similar proportion in both groups before and after the treatment (*p* =  0.11, *p* =  1, respectively).

**Conclusions:**

Olfactory training alone and olfactory training with associated with melatonin, multivitamins and topical sodium citrate did not show significant differences in improving olfactory function in post-COVID-19 patients.

**Level of evidence:**

3.

## Highlights

• The study evaluated adding melatonin, multivitamins and sodium citrate to olfactory training for COVID-19-related smell loss.
• No significant difference was found between olfactory training alone and with the additional supplements.
• Both approaches showed similar improvements in smell function and reported symptoms.

## Introduction

Among the challenges in the clinical management of olfactory dysfunction, the lack of understanding of the olfactory system and the lack of available therapeutic options stand out.^1^ Although several treatments have been tried for olfactory dysfunction, such as corticosteroids and vitamin A, no highly effective pharmacological therapy has yet been established. Among non-pharmacological therapies, olfactory training appears as a promising treatment option.^2^ Repetitive and controlled exposure to specific odors has been shown to promote improvement in olfactory function for patients with post-infectious, post-traumatic, and idiopathic causes of olfactory dysfunction, with level 1a evidence supporting its efficacy.^3^, ^4^, ^5^ The mechanism underlying the improvement in olfactory function with olfactory training is closely linked to the regenerative capacity of the olfactory mucosa and the neuromodulation of olfactory sensitivity.^6^ Evidence suggests that olfactory training induces plasticity in neural circuits, and may even lead to an increase in the olfactory bulb volume in trained patients.^7^ However, it should be noted that olfactory training requires a prolonged treatment duration, ranging from 3 months to 2 years, which may pose challenges to patient adherence.^8^ Hence, complementary therapies that can enhance the effectiveness of treatment within a shorter timeframe are crucial for achieving better outcomes.

Melatonin, zinc, and sodium citrate are drugs that have been proposed to potentially restore olfactory function. Melatonin has been shown to have anti-inflammatory and antioxidant effects, protecting neurons and promoting functional neurological recovery in animal model of nervous tissue injuries.^9^, ^10^ Due to its activity in regulating the immune system and oxidative stress and its anti-inflammatory action, melatonin has the potential to treat olfactory loss caused by COVID-19 and other viral infections, but studies need to be done to prove its effectiveness.^11^ Zinc salts have also been considered effective in the treatment of sensorineural olfactory loss in patients with post-head trauma smell loss, possibly through mechanisms related to olfactory receptor cell regeneration.^12^ In addition, the recovery of the olfactory function was faster in patients que who received zinc therapy^13^ and a decrease in zinc in the saliva of COVID-19 patients with dysgeusia was found.^14^ Sodium citrate, on the other hand, has shown promising results in improving sense of smell in patients with non-conductive causes of smell loss possibly by reducing mucosal Ca^2+^ levels and negative feedback in the olfactory pathway, potentially increasing excitability of olfactory neurons.^1^

Given the need for further investigation into these medications, the search for improved clinical management of olfactory dysfunction, and the high prevalence of smell loss, this study aimed to investigate the effects of concurrent administration of melatonin, sodium citrate, and multivitamin with zinc, along with olfactory training, for the treatment of olfactory loss in patients with varying degrees of loss caused by COVID-19 and other upper airway infections. This study is the first to examine the potential benefits of this combination therapy in such patients.

## Methods

### Subjects and study design

In this study, we conducted an analysis of medical records of patients aged 14–75 years who experienced olfactory loss after upper airway infection, including COVID-19 (as shown in Table 1). They were attended at a Smell and Taste Clinic between August 2015 and September 2022. We excluded patients with chronic rhinosinusitis, previous cranioencephalic trauma, smoking history, previous nasal surgery, use of melatonin, sodium citrate, zinc, or medication for psychiatric diseases (excluding depression).

**Table 1:** Baseline characteristics of study population.

Variable	Control (*n* = 33)	Treatment (*n* = 33)	*p*-value
Age, mean in years (SD)	44.5 (17.1)	40.9 (15.6)	0.4
Sex, *n* (% male)	16 (49)	16 (49)	1
Race, *n* (% white)	27 (82)	24 (73)	0.6
Olfactory loss time in months, mean (SD)	7.1 (7.4)	10.8 (8.7)	**<0.01**
COVID-19, *n* (%)	24 (73)	22 (67)	0.8
UPSIT ® scores, mean (SD)	20.3 (7.7)	20.9 (7.6)	0.76
Subjective olfactory score^a^, mean (SD)	3.8 (3.4)	4.1 (2.2)	0.37
Level of discomfort score^a^, mean (SD)	6.7 (3.9)	7 (3.2)	0.88

SD, Standard Deviation.

a Self-reported perception using a scale of 0–10 points, with 0 indicating a complete lack of sense of smell and 10 indicating normal sense of smell. In addition, the level of discomfort caused by the loss of smell was evaluated using a similar scale, with 0 indicating no discomfort and 10 indicating extreme discomfort. Statistically significant p-values are indicated in bold font.

We evaluated two groups of patients, matched for age and sex: the control group, who received olfactory training only, and the treatment group, who received olfactory training along with melatonin, sodium citrate, and multivitamins with zinc (Centrum® from A to Z). Patients who underwent treatment for 3 months were selected. We assessed olfactory function before and after treatment using an olfactory test and self-report. The study followed the STROBE criteria. The project was approved by the ethics committee for research involving human from the State University of Londrina beings and all participants signed an informed consent form.

### Olfactory training

The olfactory training for patients involved sniffing four odors (phenylethyl alcohol, eugenol, citronellal, and eucalyptol) twice a day for 10 s over a period of 3 months, as described in previous studies.^3^, ^8^

### Treatment

Patients were prescribed 5 mg of melatonin to be taken at night, one tablet of a vitamin-mineral supplement (Centrum®), which contained 7 mg of zinc per tablet according to the package leaflet and other components described in Supplementary Table 1, to be taken every 12 h, and one jet of 9% sodium citrate to be applied to each nostril in the direction of the olfactory fossa one hour before lunch and dinner, for a continuous period of 90 days.

### Olfactory test

To evaluate the effectiveness of the study intervention, we considered a significant improvement to be an increase of 4 points or more on the University of Pennsylvania Smell Identification Test (UPSIT®) after 3 months of therapy.^15^ The UPSIT® is composed of four booklets, each containing 10 odors presented one per page. The odors are encapsulated in plastic microcapsules located in a brown band at the bottom of each page. Patients are instructed to scratch this band with a pencil to release the odor and then select the corresponding multiple-choice description. Based on the UPSIT® score, we classified patients' olfactory function as normosmia (above 31 for men and above 34 for women), microsmia (between 17 and 31 for men and 19 and 34 for women), or anosmia (below 17 for men and 19 for women).^10^ Additionally, we evaluated patients' olfactory function based on their self-reported perception using a scale of 0–10 points (0 indicating no sense of smell and 10 indicating normal sense of smell) and their level of discomfort caused by the loss of smell using a similar scale (0 indicating no discomfort and 10 indicating extreme discomfort). We also inquired about any qualitative complaints of parosmia (distorted sense of smell in which a smell is perceived as something else) and phantosmia (perception of odor in the absence of an actual stimulus).

### Statistical analysis

The sample size was determined by detecting a significant difference of 4 points in the olfactory test, using an alpha level of 5%, beta level of 20%, and a standard deviation of 5 points. Ultimately, each group must contain at least 26 participants. Statistical analyses were performed using STATA software version 16.0 (StataCorp LP, College Station, Texas, USA). A logistic regression model was employed to examine the improvement on the olfactory test between groups while controlling for the slight variation in the time of olfactory loss onset among them. The *t*-test for independent samples was used to compare the two groups, while the Mann-Whitney test was utilized for variables that did not exhibit normal data distribution. Fisher's exact test was employed for the analysis of categorical variables. These tests were conducted to evaluate the efficacy of the treatment and to establish the significance of the results.

## Results

### Population

A total of 66 patients were included in the study, with 33 patients receiving olfactory training alone and 33 patients receiving olfactory training along with medications. The percentage of patients diagnosed with post-COVID-19 olfactory loss, sex and race was comparable between the two groups. The treatment group had a significantly longer duration of olfactory loss than the control group (Table 1). Among the reported adverse effects, 5 patients (15%) reported experiencing nasal burning following the use of sodium citrate.

### Treatment effectiveness

**Figure 1:**
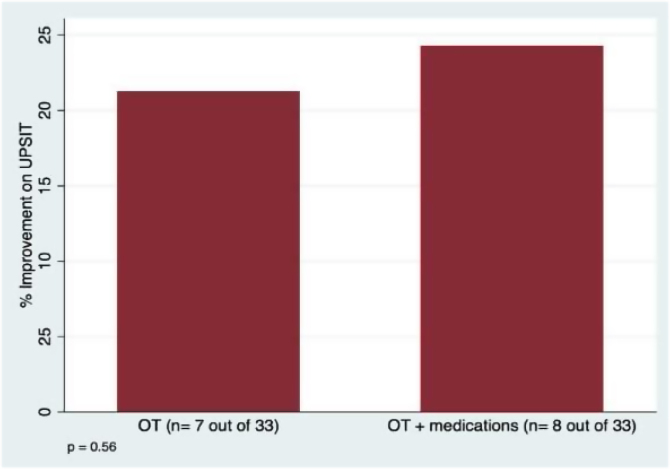
Proportion of participants with clinically significant improvement on UPSIT scores (4 or more points) after Olfactory Training (OT) alone or with the melatonin, multivitamin and sodium citrate treatment.

The results showed that 78.8% of participants in the olfactory training alone group did not experience any improvement in UPSIT^Ò^ scores, while only 21.2% (*n* = 7) showed improvement. In contrast, 75.8% of participants in the olfactory training with medications group did not experience any improvement in UPSIT^Ò^ scores, while 24.2% (*n* = 8) showed improvement (Fig. 1). The logistic regression controlling for the time of olfactory loss revealed no significant difference in the proportion of participants who showed improvement in UPSIT^Ò^ scores between the olfactory training alone group and the olfactory training with medications group (OR = 1.43, 95% CI 0.43–4.8, *p* =  0.56).

The distribution of patients across olfactory ability categories before and after treatment is presented in Table 2. There was also no significant difference in the improvement in olfactory loss categories (anosmia, microsmia, or normosmia) between the groups (*p* =  0.84). In the intervention group, 36.4% had improved from anosmia to hyposmia on the UPSIT^Ò^, compared to 28.6% in the control group. Three patients in total had improved to normosmia, with two in the medication group.

**Table 2:** Comparison of UPSIT ® categories before and 3-months after treatment with olfactory training alone (controls) or sodium citrate, melatonin, and multivitamin supplements with zinc more olfactory training.

Controls *n* (%)			
Before		After	
Anosmia	14 (42.4)	Anosmia	10 (71.4)
		Microsmia	4 (28.6)
		Normosmia	0
Microsmia	19 (57.6)	Anosmia	1 (5.3)
		Microsmia	17 (89.4)
		Normosmia	1 (5.3)

Treatment *n* (%)			
Before		After	
Anosmia	11 (33.3)	Anosmia	7 (63.6)
		Microsmia	4 (36.4)
		Normosmia	0
Microsmia	22 (66.7)	Anosmia	2 (9.1)
		Microsmia	18 (81.8)
		Normosmia	2 (9.1)

Both groups showed improvement in their average test scores after three months, however the average score on UPSIT^Ò^ was similar between the groups at baseline and at the three-month follow-up (Table 3). Regarding self-reported olfactory function and level of discomfort with the olfactory loss, there was also no difference. For self-reported olfactory ability, 64% of participants in both the control and treated groups noticed some improvement in smell, and this difference was not statistically significant (*p* =  1). For discomfort associated with loss of smell, 33% of participants in the control group and 36% in the treated group reported a decrease in discomfort, but this difference was also not statistically significant (*p* =  1).

**Table 3:** Comparison of olfactory evaluation values before and after treatment.

UPSIT ® scores, mean (SD)	Baseline	3-month	Change** (95% CI)	*p*-value
Controls	20.3 (7.7)	21.6 (8)	Reference	Reference
Treatment	20.9 (7.6)	22.8 (6.5)	+0.93 (−1 – 2.9)	0.34

Subjective olfactory score*, mean (SD)	Baseline	3-month	Change (95% CI)	*p-*value
Controls	3.8 (3.4)	5.3 (3.3)	Reference	Reference
Treatment	4.1 (2.2)	5.5 (2.4)	+0.1 (−0.8 – 1.1)	0.76

Level of discomfort score**, mean (SD)	Baseline	3-month	Change (95% CI)	*p-*value
Controls	6.7 (3.9)	5.7 (3.9)	Reference	Reference
Treatment	7 (3.2)	6.4 (3.1)	+0.1 (−1.5 ‒ 1.6)	0.94

* self-reported perception using a scale of 0---10 points (0 indicating no sense of smell and 10 indicating normal sense of smell) and their level of discomfort caused by the loss of smell using a similar scale (0 indicating no discomfort and 10 indicating extreme discomfort).

** Level of discomfort caused by the loss of smell (0 indicating no discomfort and 10 indicating extreme discomfort).

The results related to qualitative complaints showed that among the 33 patients in the study, 12 patients in the control group (36.4%) reported parosmia at the first visit, whereas 16 patients in the medication group (48.5%) reported the same symptom. Improvement was observed in 50% of patients in the control group, while only 17.6% of patients in the medication group showed improvement, although this difference was not statistically significant (*p* =  0.11). Phantosmia was reported by 6 patients in the control group (18.2%) and 8 patients in the medication group (24.2%). After three months, the improvement rates were 66.7% in the control group and 62.5% in the medication group, with no statistically significant difference (*p* = 1).

## Discussion

In this analysis of medical records, we evaluated the effectiveness of olfactory training alone versus olfactory training combined with melatonin, sodium citrate, and multivitamins with zinc for the treatment of olfactory loss in patients who experienced upper airway infections, including COVID-19. Our results showed that while both groups had a similar percentage of patients who did not experience any improvement in UPSIT® scores, the group receiving the additional medications showed a slightly higher percentage of patients who showed improvement in UPSIT® scores compared to the olfactory training alone group.

The use of olfactory training as a treatment for olfactory loss has been studied before, and our findings are consistent with previous studies that have shown mixed results in terms of its effectiveness.^3^, ^8^ However, our study adds to the literature by examining the potential additional benefits of combining olfactory training with melatonin, sodium citrate, and multivitamins with zinc.

The qualitative complaints result of the study highlight the prevalence of parosmia and phantosmia in the patient population, with higher reported rates in the medication group compared to the control group. However, the findings regarding improvement rates reveal an interesting trend, with a higher percentage of patients in the control group showing improvement compared to the medication group, although the difference did not reach statistical significance. It is important to consider the small sample size of the study, which may have limited the statistical power to detect significant differences.

Melatonin is a hormone that has been shown to have antioxidant and anti-inflammatory properties,^9^ and it has been suggested that it may have a protective effect on the olfactory epithelium and potentially aid in the recovery of olfactory function. Sodium citrate has been used as improve sense of smell in patients with non-conductive causes of smell loss.^1^ Zinc is an essential mineral that has been shown to play a role in immune function and wound healing, and it has been suggested that it may have a potential role in the recovery of olfactory function.^12^

The mechanism of post-COVID-19 olfactory loss is not yet fully elucidated, but data implicate inflammation, with or without infection, in the olfactory epithelium, olfactory bulb, or both.^16^ Therefore, the study of new therapies with anti-inflammatory medications could help in the correct regeneration of the olfactory epithelium.

Our study has some limitations that should be considered when interpreting the results. First, the retrospective design of the study may have introduced selection bias and confounding variables, and the lack of randomization may have affected the comparability of the two groups. Additionally, the small sample size may have limited the power of the study to detect significant differences between the groups.

Despite these limitations, our study provides preliminary evidence that combining olfactory training with melatonin, sodium citrate, and multivitamins with zinc may result in a slightly higher percentage of patients showing improvement in UPSIT^Ò^ scores compared to olfactory training alone. Further research with larger sample sizes, randomized controlled trials, and longer follow-up periods are needed to confirm these findings and to better understand the potential benefits of these medications in the treatment of olfactory loss. Although the study did not show a significant improvement in olfactory function with treatment, its results can help in the treatment of individuals with the disease, avoiding the use of inefficient medications, reducing the inappropriate use of medications and possible side effects, and reducing treatment costs.

A recently published study conducted with patients with post-COVID olfactory loss used treatment with olfactory training and adjuvant (palmitoylethanolamide co-ultra-micronized with luteolin -one-PEA-LUT, an anti-neuroinflammatory supplement) evaluated with another olfactory test (Sniffin' Sticks) showed an improvement in the sense of smell with the combined use of these therapies.^17^ This shows the importance of studies in this regard.

Our study demonstrates that combining olfactory training with melatonin, sodium citrate, and multivitamins with zinc does not differ significantly from using olfactory training alone. Therefore, the use of these medications for treatment of olfactory loss in patients who have experienced upper airway infections, including COVID-19 is not suggested. Further research is warranted to confirm these findings and to elucidate the underlying mechanisms of action of these medications in promoting recovery of olfactory function. Clinicians should consider the potential benefits and risks of using these medications in conjunction with olfactory training when developing treatment plans for patients with olfactory loss.

## Conclusion

Olfactory training alone and olfactory training with medications (sodium citrate, melatonin, and multivitamin supplements with zinc) did not show significant differences in improving olfactory function in post-COVID-19 patients, as measured by UPSIT® scores, self-reported olfactory ability, discomfort with olfactory loss, and qualitative symptoms. Further research with larger sample sizes may be warranted to confirm these findings.

## Authors’ contributions

Ellen Cristine Duarte Garcia: Conducted the data collection; Conducted the analyses; drafted the initial version; and Revised the manuscript.

Letícia Ribeiro Rosa: Conducted the data collection; Drafted the initial version; and Revised the manuscript.

Ana Caroline Rodrigues dos Santos: Conducted the data collection; Drafted the initial version; and Revised the manuscript.

Gabrieli Kaori Alves Ishimatsu: Conducted the data collection; Drafted the initial version; and Revised the manuscript.

Natália Medeiros Dias Lopes: Conducted the data collection; Drafted the initial version; and Revised the manuscript.

Marco Aurélio Fornazieri: Conceptualized and designed the study; Conducted the analyses; Drafted the initial version; and Revised the manuscript.

All authors approved the final manuscript as submitted.

## Conflicts of interest

The authors declare no conflicts of interest.
