# Supplementary material for: Investigating the efficacy of melatonin, topical sodium citrate, and multivitamin with zinc as a potential treatment for postinfectious loss of smell
Source: Braz J Otorhinolaryngol. 2024 Aug 31;90(6):101496. doi: 10.1016/j.bjorl.2024.101496 (PMC11409182; doi:10.1016/j.bjorl.2024.101496)
Supplement: Supplementary file 1 [file mmc1.docx]

**Supplementary Table 1** Centrum^Ò^ nutritional information.

| **Amount per serving of 1.4g (1 tablet)** | | **%DV** |
| --- | --- | --- |
| Carbohydrates | 0.2g of which | 0.1 |
| Sugars | 0g | 0 |
| Total fat | 0g | 0 |
| Saturated fat | 0g | 0 |
| Trans fat | 0g | 0 |
| Monounsaturated fat | 0g | 0 |
| Polyunsaturated fat | 0g | 0 |
| Cholesterol | 0 mg | 0 |
| Vitamin A | 200.00 mcg | 33 |
| Vitamin D | 10.00 mcg | 200 |
| Vitamin C | 60 mg | 133 |
| Vitamin E | 7 mg | 70 |
| Thiamin | 1.6 mg | 133 |
| Riboflavin | 2.2 mg | 169 |
| Niacin | 20 mg | 125 |
| Vitamin B6 | 2.6 mg | 200 |
| Folic acid | 500.00 mcg | 125 |
| Vitamin 12 | 7.22 mcg | 300 |
| Biotin | 30.00 mcg | 100 |
| Pantothenic acid | 4.7 mcg | 94 |
| Vitamin K | 65.00 mcg | 100 |
| Calcium | 250 mcg | 25 |
| Iron | 8.1 mg | 58 |
| Magnesium | 100 mg | 38 |
| Zinc | 7 mg | 100 |
| Iodine | 32.50 mcg | 25 |
| Copper | 450.00 mcg | 50 |
| Selenium | 34.00 mcg | 100 |
| Molybdenum | 22.50 mcg | 50 |
| Chromium | 17.50 mcg | 50 |
| Manganese | 1.2 mg | 52 |
| Phosphorus | 125 mg | 18 |
| “Not a significant source of energy value, protein, dietary fiber, and sodium”. | | |

DV, Daily Value.
